# Migrants and health care

**DOI:** 10.2471/BLT.22.020322

**Published:** 2022-03-01

## Abstract

On the eve of a high-level meeting on health and migration in Europe, attention is focusing on the need for collaborative action to support rights-based health service provision. Andréia Azevedo Soares reports.

Team leader at the Swedish Red Cross unit in Stockholm, Sweden, Lau Dahlgren is used to seeing patients in distress. “Most of the people who come in here are from former Soviet Union countries, but we also see people from Latin America and Africa. Many come in with health problems, but they can also be struggling with accommodation, where to get their next meal and simply making themselves understood,” he says.

The patients Dahlgren sees are a mix of forced and economic migrants, the former being a term used for people who have been compelled by circumstances to leave their home country, whether those circumstances derive from natural causes such as storms and floods or man-made causes such as military conflict or political persecution.

Dahlgren and his team of three nurses and a social worker offer basic health care including a medical examination and medication where appropriate, but if they need to refer patients for more complex care they come up against a problem.

“Many of the people we see are undocumented and they are reluctant to enter into contact with the health system, but even people with a permit have problems,” he says, noting that access to Sweden’s health-care system requires a social security number, which is generally only granted to migrants holding permits valid for more than a year. “People with a permit to stay for less than one year only have access to emergency health care and must pay the full cost,” Dahlgren says. “To see a doctor costs from Euro 200 (US$ 223), while care for non-complicated childbirth is Euro 3000 (US$ 3350).”

Like many European countries, Sweden has hardened its policies towards immigrants in recent years, including policies concerning access to health services. It is a policy shift that reflects a fundamental change in public perceptions according to Dahlgren.

Observable in many European countries, that change is often explained in terms of the so-called “migrant crisis” of 2015, in which some 1.3 million people entered Europe requesting asylum, many of them from the war in the Syrian Arab Republic.

“The flows of refugees and migrants that Europe experienced in 2015 not only put the issue of migration at the top of the political agenda in many countries, it also highlighted shortcomings in health system responses,” says Dr Santino Severoni, Director of WHO’s Health and Migration Programme.

In Severoni’s assessment, the default response was to treat the challenge as an emergency. “Policy-makers often tried to adopt temporary solutions, rather than addressing long-term malfunctions, and strengthening health system capacity.” he says.

“Talking about migrant health is not enough.”

Jasmijn Slootjes

However, as Severoni points out, the flow of forced migrants into the region had been steadily increasing since the late 2000s, making the 2015 crisis part of a continuous flow rather than a unique event.

To support what Severoni and his colleagues at WHO viewed as urgently needed systemic reform, the WHO Regional Office for Europe drew up an action plan (*The Strategy and action plan for refugee and migrant health in the WHO European Region*) which was unanimously endorsed by the WHO Regional Committee for Europe in September 2016.

The nine-point plan emphasized the need to overcome formal and informal barriers to health care, including regulations in certain countries that determine access to services. It also called for the health needs of refugees and migrants to be met through “existing national health structures in accordance with national legislation and policies”.

For Severoni, the endorsement of the strategy represented a watershed moment. “Bringing health issues and more particularly the question of health service provision into discussions about the broader response to the migrant issue back then was historical,” he says.

He notes, however, that despite that endorsement there has been a lack of concrete action on any of the key recommendations since 2016. It is Severoni’s hope that the high-level meeting of health ministers and representatives of the 53 Member States of the WHO European Region that is to be held between 17 and 18 March 2022, in Istanbul, Turkey, will mark a new departure in the efforts to address the challenges faced.

“With the European Action Plan coming to a close we have an important opportunity to reflect on past experience and lessons learned,” he says.

Jasmijn Slootjes, Senior Policy Analyst at the Brussels-based Migration Policy Institute Europe is also looking forward to the meeting, but she stresses the need for action. “Progress has been made, with important pacts on migration mentioning health issues and research interest increasing dramatically. Member States and the European Commission are acknowledging the importance of migration and health policies and have shown a readiness to discuss the subject but talking about migrant health is not enough. Now we need to move forward.”

Key to moving forward, in Slootjes’ view, is making sure that policy-makers have all the information and guidance they need. “There's been so much research about best practices and how to overcome barriers to migrants accessing health care, but there’s an implementation gap. Policy-makers can’t find these best practices. They exist out there in academic literature, but we need to get all that information to the right people. WHO has an important role to play here.”

As Severoni points out, WHO has in fact generated a considerable body of guidance, including two fully developed action plans: the European plan already mentioned; and the 2019 WHO global action plan*: Promoting the health of refugees and migrants*, which was developed in close collaboration with the International Organization for Migration and the United Nations High Commissioner for Refugees.

WHO also issued a multi-country review of health systems' responsiveness to refugee and migrant health needs, and a review of the literature covering the common health needs of refugees and migrants. Most recently, WHO released *Refugee and migrant health: Global Competency Standards for health workers* to support a competency-based outcomes approach to education and training for health workers who provide services to refugees and migrants.

For Professor Hannah Bradby, head of the Sociology Department at Uppsala University, there is also a need for new thinking on migrant health service provision, taking full account of the complex social determinants of health that are an inherent part of the migrant experience.

“Conventional forms of health service delivery […] will not be addressing [migrants’] needs.”

Hannah Bradby

“We need to acknowledge the complex situations in which migrants often find themselves and be ready to meet their needs through integrated health and social care. In many cases their primary needs may not even be health care. Unmet health needs are not always for antibiotics or a doctor's appointment. In some cases, migrants are more concerned about food or personal hygiene or shelter. If we stick to conventional forms of health service delivery, we will not be addressing these needs,” she says.

Bradby believes that nongovernmental organizations (NGOs) working with migrants have lessons to share in this regard, partly because they have close contact with migrant populations in the field. “Our experience in 2015 and 2016 in the eastern Mediterranean and in the Balkans demonstrated that NGOs were much better at going into the places where migrants were and connecting with them,” she says.

Bradby also believes that NGOs have a key role to play in supporting the development of whole-of-route strategies and in delivering health services in destination countries. “NGOs are often known to migrants from their experience on the road or in their departure countries. And because they are not connected with official authorities, migrants tend to feel safe when accessing the health services that they provide.”

Whatever the approaches taken in ensuring access to health services for migrants, it is clear that a fundamental prerequisite of access is acknowledging their right to those services and making sure that right is respected. “Every time access to health is denied based on a migrant's citizenship we observe the emergence of a new form of inequality,” says Bradby.

It is an inequality that Lau Dahlgren confronts every day. “My hope is that health professionals will one day be allowed to forget about patients’ legal status and that they can give them what they need regardless of their papers,” he says.

As 53 European Member States prepare to discuss the drawing up of a new action plan in Istanbul, migrants continue to arrive. “Europe currently hosts around a third of the global international migrant population which is estimated to be 281 million people,” says WHO’s Severoni. “That population is expected to swell in the coming years. It is not a population we can simply ignore.”

**Figure:**
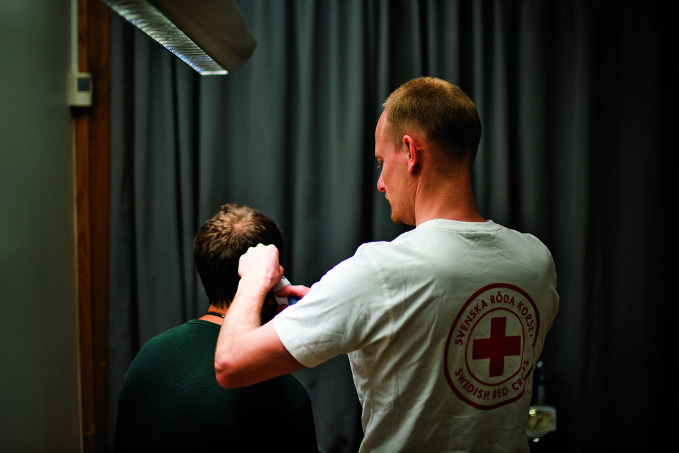
Examining a patient at the Swedish Red Cross referral unit in Stockholm, Sweden.

Marie Sparréus/Swedish Red Cross

**Figure:**
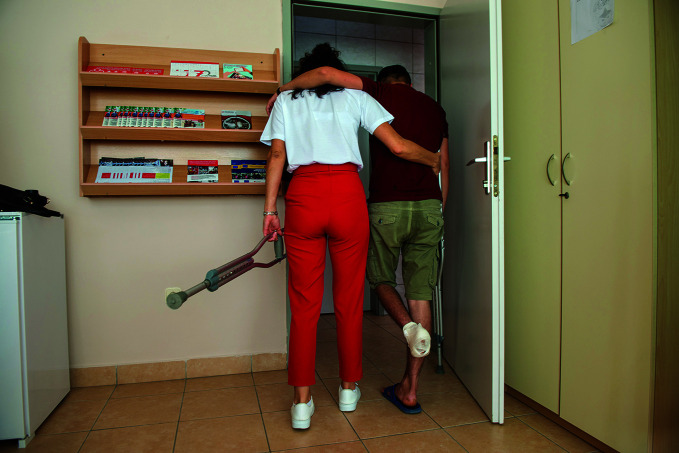
A doctor in Skopje, North Macedonia helps a young migrant from Afghanistan who lost his foot jumping from a moving train.

Victor Lacken/IFRC

